# Brazilian Gold Miners Working Irregularly in French Guiana: Health Status and Risk Determinants

**DOI:** 10.3390/tropicalmed10010012

**Published:** 2024-12-31

**Authors:** Amanda Figueira da Silva, Vivian da Cruz Franco, Maylis Douine, Hermano Gomes Albuquerque, Louise Hureau, Alice Sanna, Pamela Mosquera Atehortua, Rafael dos Santos Pereira, Simone da Silva Santos, Paola Barbosa Marchesini, Gustavo Bretas, Margarete do Socorro Mendonça Gomes, Yann Lambert, Martha Cecília Suárez-Mutis

**Affiliations:** 1 Laboratory of Parasitic Diseases, Oswaldo Cruz Institute/Fiocruz, Rio de Janeiro 21040-360, Brazilhermanofio@gmail.com (H.G.A.); simonesantosbio@gmail.com (S.d.S.S.);; 2 Post-Graduate Program in Tropical Medicine, Oswaldo Cruz Institute/Fiocruz, Rio de Janeiro 21040-360, Brazil; 3 Andréé Rosemon Hospital (CHAR), Cayenne 97306, French Guiana; maylis.douine@ch-cayenne.fr (M.D.);; 4 Ministry of Health, Department of Transmissible Diseases Surveillance, Brasilia 70058-900, Brazil; 5 Independent Researcher, Rio de Janeiro 22250-040, Brazil; 6 Health Surveillance Superintendent of Amapá, Amapá 68902-865, Brazil

**Keywords:** gold miners, malaria, health determinants

## Abstract

Brazilian garimpeiros are a highly hard-to-reach and mobile population, with little access to basic hygiene and health services, and have been crossing the border to work irregularly in gold mines in French Guiana since the 1990s. This study aimed to characterize this population and identify their main health problems. A cross-sectional study was carried out in the municipality of Oiapoque-AP, with two surveys: before (2018) and after (2019) the implementation of Malakit. Individuals were recruited from resting places and given a questionnaire regarding demographic variables, history and knowledge of malaria, and health issues in the mines. Simultaneously, a clinical evaluation was performed. The garimpeiros were mainly men from Maranhão, Pará, and Amapá, with a low educational level and who had worked on average for 10 years in the mining sites. The study population mentioned numerous health problems: malaria, followed by leishmaniasis, flu, body aches, headaches, and digestive problems. Other diseases mentioned were skin diseases, bat bites, hepatitis, and HIV infection. This vulnerable population is constantly subjected to heavy routines and exposed to different diseases and infections that can spread across borders. Knowledge of this is essential for developing public health policies that can be integrated into specific epidemiological situations.

## 1. Introduction

Artisanal Brazilian gold miners (also known as garimpeiros) cross the border of French Guiana (FG) to work at irregular gold mining sites [1,2]. It is estimated that approximately 11,000 individuals in at least 600 gold mines within the French Amazon rainforest live in remote and difficult-to-access areas [3]. According to the World Wildlife Fund France, it is estimated that for every 1 or 2 tons of gold mined legally, up to 10 tons of gold are mined irregularly [4]. Furthermore, approximately 2.000 individuals enter the mines in Suriname and French Guiana each year for the first time [3]. In Zimbabwe, some factors point to the undeniable vulnerability of these individuals: living in remote locations, with little access to primary hygiene conditions, basic sanitation, or quality water, as well as little access to health services, among others [5]. The same irregular work situation in French Guiana hinders health services, although emergency services are accessible at hospitals and health centers in the region [6]. A study conducted in 2015 assessed the health conditions of these workers on the Suriname–French Guiana border and identified poor health status with infectious and non-infectious diseases, with malaria being the main grievance on this frontier [7]. Between 2018 and 2020, the Malakit project was developed as a population-based intervention in which kits for self-diagnosis and proper self-treatment, along with health education and information activities, were delivered to gold miners with symptoms of malaria inside the mines [8]. The strategy used to find miners was to actively search for resting sites on the international border side, where they go to replenish supplies, seek medical help, rest, exchange gold, or leave the mining site. The Brazilian municipality of Oiapoque, located on the border between Brazil and French Guiana, has several sites that shelter miners and serve as a pole of attraction for individuals from various states seeking to work in the Guiana Shield mines [9]. The health status of miners who cross this frontier through Oiapoque is unknown, and this information is vital to support health services taking action to improve the health status of these populations and their surroundings. This study aimed to characterize the population of miners who work irregularly in the mines of French Guiana and identify the main health problems of this population.

## 2. Materials and Methods

### 2.1. Study Design

Two cross-sectional studies were conducted before and after implementing the Malakit Project, an interventional study intended to target *P. falciparum* malaria reservoirs in a population of undocumented miners working in French Guiana [10]. Participants were recruited from the resting sites of miners in the municipality of Oiapoque, Amapá, on the border between Brazil and French Guiana.

### 2.2. Study Sites

**Figure 1:**
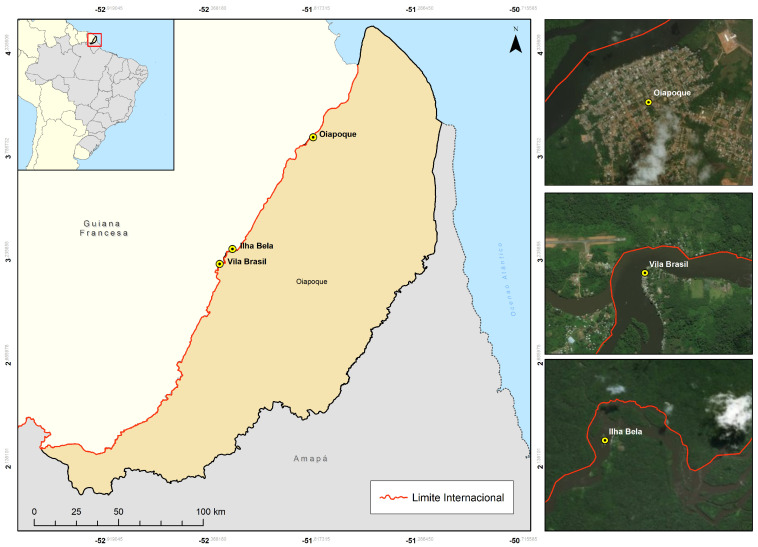
Study sites in the municipality of Oiapoque—AP, Brazil.

The Brazil–Guiana transboundary zone (ZTBG) is 730 km long and comprises two municipalities on the Brazilian side: Oiapoque and Laranjal do Jari. On the other side of the border is French Guiana, an overseas department of France with three communes on the border: Saint-Georges de L’Oyapoque, Camopi, and Ouanary. The study was conducted in the municipality of Oiapoque, located in the northern part of the state of Amapá, with an extension of 22,625 km^2^, with an estimated population of 27,270 inhabitants, and a hot and humid equatorial climate [11]. Despite migration restrictions, Oiapoque is a vital stepping-stone to the French Guiana and Suriname mining sites. Three locations were determined for the recruitment of the participants: the city of Oiapoque (Latitude: 3°49′53″ North, Longitude: 51°50′7″ West) is considered a twin city, separated from Saint-Georges (French Guiana) by the Oiapoque river; Ilha Bela (Latitude: 3°15′15.15″ North, Longitude: 52°15′24.54″ West) is a settlement of about 200 houses located five hours by boat from Oiapoque; Vila Brasil (Latitude: 3°10′14.30″ North, Longitude: 52°19′48.69″ West), situated seven hours from the city by the Oiapoque river, is a small village with approximately ninety houses [12]. Ilha Bela and Vila Brasil are key transit locations for the mines in the west of French Guiana and close to the mines near Suriname. Both locations survive by selling supplies to the miners (Figure 1).

### 2.3. Study Population and Recruitment Strategy

The study population consisted of persons engaged in irregular gold mining extraction in areas of French Guiana and people who lived in or visited these mines for various activities, such as sex workers, cooks, and traders, among others, all of whom will be referred to in this study as “garimpeiros”. Due to the irregular conditions of stay in the French territory, two recruitment strategies were used. In the town of Oiapoque (an urban area), individuals were recruited at hotels and guesthouses, where gold miners stayed temporarily to exchange gold, buy supplies, rest, or treat illnesses. The second strategy was recruitment in Ilha Bela and Vila Brasil, which were used as stepping-stones to the city or another mining site, where the miners stayed for a few hours or days. When gold miners arrived on their trips, they were approached to participate in the study. Oiapoque, Vila Brasil, and Ilha Bela were called “resting places”. A convenience sampling was performed using the “encounter opportunities” method with an adaptation of the snowball sampling technique, which comprises approaching a person who indicates another person and so on [13].

### 2.4. Procedures

Once the miners were identified, they were invited to participate in the research after being informed about the objectives and procedures that would be carried out. Miners were only included after being declared sufficiently informed and signing an informed consent form (ICF). Inclusion criteria included working or frequently visiting mines in French Guiana, being at rest sites for less than seven days before recruitment, being 16 years of age or older, and signing the ICF. Garimpeiros that proceeded from Brazilian mines were excluded. Miners interviewed in the second sectional study were excluded if they were included in the first study to avoid repetition of data. A structured questionnaire was administered to collect sociodemographic data, routine characteristics, and health history. A team physician also performed a physical examination, and samples were collected for thick smears and laboratory tests.

### 2.5. Sample Collection and Processing

Thick blood smears for malaria were collected for microscopy, and individuals who tested positive were treated using the malaria regimens recommended by the Brazilian Ministry of Health [14]. Subsequently, 5 mL of whole blood was collected from everyone included in the study in a BD Vacutainer^®^ tube with EDTA (AB Medical, Inc.^®^, BD Vacutainer, São Paulo, SP, Brazil) for performing diagnostic PCR of *Plasmodium* spp.—following the Snounou protocol [15].

### 2.6. Variables

A single physician performed all clinical examinations during the first cross-sectional study, and two examined the individuals during the second study. Individuals with an axillary temperature ≥ 38 °C were considered febrile. Blood pressure was measured after 15 min of rest, in a sitting position, using a digital sphygmomanometer, considering normal values for the systolic pressure (SBP) < 140 mmHg and the diastolic pressure (DBP) < 90 mmHg, Stage 1 hypertension (SBP: 140–159, DBP: 90–99), Stage 2 hypertension (SBP: 160–179, DBP: 100–109), Stage 3 hypertension (SBP: ≥180 and DBP: ≥110), and isolated systolic hypertension (SBP ≥ 140 and DBP < 90); (Brazilian Hypertension Guidelines 2010). Body mass index (BMI, weight/h^2^) was calculated, classifying as underweight a BMI of <18.5, normal (18.5–24.9), overweight (25–29.9), obese (30–39.9), and severe obesity (≥40). Mucocutaneous jaundice, cardiac auscultation, splenomegaly, lower-limb edema, and skin lesions were checked. Splenomegaly was assessed using Hackett’s index: 0 (no splenomegaly), 1 (at forced inspiration), 2 (above half the distance between the costal margin and the umbilicus), and 3 (below half the distance between the costal margin and the umbilicus [16].

### 2.7. Data Analysis

The data were collected using structured questionnaires. All data were stored and analyzed using the Epi Info™ 7.2.1.0 software (CDC) and GraphPad Prism 8 (GraphPad Software Inc., GraphPad Software, Boston, Massachusetts, the United States). These data were used for descriptive analyses using significant variables such as age, sex, location attended, and length of stay at the mining site. Chi-square was used for categorical variables. A 95% confidence interval was established, and a *p*-value of less than 0.05 was used as the significance level. For the maps, we considered three levels of intensity of the migratory flows: low (0.1–10.0%), medium (10.1–19.9%), and high (above or equal to 20%). Three levels were also considered to define the Gross Domestic Product of each state: low (0.00–15.00), medium (15.01–30.00), and high (above 30.01). These data were obtained from the Brazilian Institute of Geography and Statistics (IBGE) [17].

## 3. Results

### 3.1. Socioeconomic Characteristics

**Figure 2:**
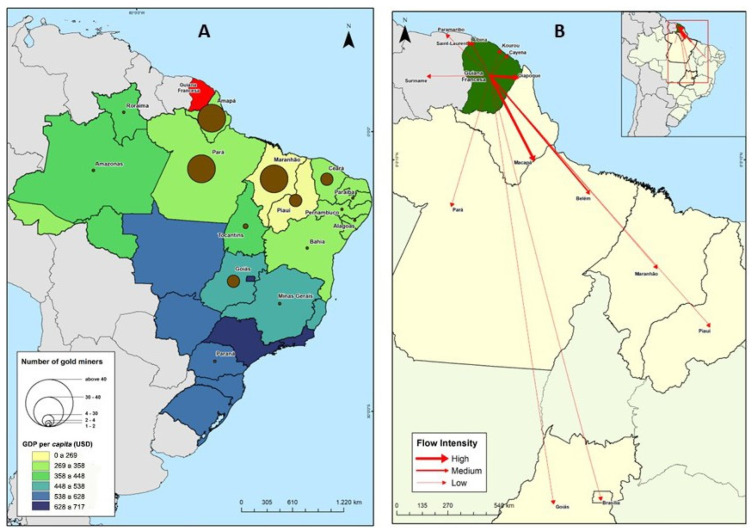
Population mobility of Brazilian miners interviewed in the municipality of Oiapoque. (**A**) State of origin of the miners and Human Development Index (HDI). (**B**) Destinations of the population of gold miners when they leave the mining site in French Guiana. Three levels of intensity were considered: low (0.1% to 10.0%); medium (10.1% to 19.9%); high (above or equal to 20%). Source: Malha Municipal 2018 (IBGE). Elaborated by: Rafael dos Santos Pereira, 2018.

A total of 292 individuals were interviewed: 174 from the first survey (2018) and 118 from the second (2019); 242 (82.9%) were in the city of Oiapoque, 49 (16.8%) in Ilha Bela, and 1 (0.3%) in Vila Brasil. Among the participants, 83.2% (243/292) were male and 16.8% (49/292) were female, of whom two (4.1%) were pregnant. The male-to-female ratio was 4.9:1. The median age was 38 years (IQR 30–48), with no difference between the sexes (*p* > 0.2) or place of inclusion (*p* = 0.22). All the interviewees were Brazilian. Although they came from 16 different Brazilian states, 87.8% proceeded from three states: 51.7% (150/290) from Maranhão, 21.7% (63/290) from Pará, and 14.5% (42/290) from Amapá; two participants did not disclose which state they were born in. Figure 2A shows the place of origin of the miners along with the Human Development Index (HDI) per state.

Among the study participants, 74.7% (218/292) had never attended school or had completed only up to the primary level of education (up to 5 years) (Table 1). Women had a significantly higher level of education (38.7%) than men (22.6%) (*p* = 0.01). It was also noted that 82.7% of the individuals from Maranhão State had no education or had only completed elementary school, and these differences were statistically significant when compared with all other individuals (*p* = 0.001).

### 3.2. Characterization of Labor at the Mines

**Figure 3:**
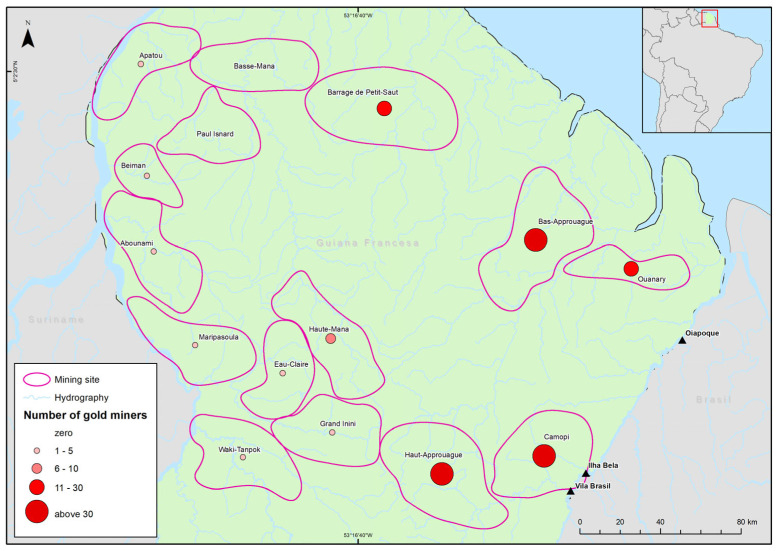
Last mining site [in French Guyana] visited by the population of garimpeiros according to geographical zone, Oiapoque-AP, Brazil.

The participants had visited approximately 60 mining sites in French Guiana over the last three years. As there were many mines, they were grouped into 15 different zones according to geographical proximity and river basin: 80.1% of the garimpeiros came from five main zones: Haut-Approuague (33.2%, 97/292), Bas-Approuague (14.0%, 41/292), Camopi (12.7%, 37/292), Barrage de Petit-Saut (10.3% 30/292), and Ouanary (9.6%, 28/292). In some cases (12.7% 37/292), it was not possible to group them into any one place, either because the name of the mine was not known or because the miners did not want to provide this information. In total, 42.3% came from four main locations: Siquini (11.7%, 34/290), Sapucaia (11.4%, 33/290), Petit Saut (9.6%, 28/290), and Ouanary (9.3%, 27/290) (Figure 3). Garimpeiros recruited at Ilha Bela or Vila Brasil came predominantly from the Haut-Approuague or Camopi mining sites, located in the western region of French Guiana, and garimpeiros recruited at Oiapoque proceeded from the Bas-Approuague, Barrage de Petit-Saut, or Ouanary zones (areas situated to the east of French Guiana and closest to Oiapoque). These differences were statistically significant (*p* < 0.001). Among the mining sites, 41.4% (118/285), 18.2% (52/285), and 17.9% (51/285) were alluvial, well, and together, respectively. Participants worked on average in 2.5 ± 2.4 mining sites over the past three years (Median 2, IQR: 1–3), ranging from 1 to a total of 15, with the majority being in French Guiana exclusively (67%) and 24% (70/291) distributed between Brazil, French Guiana, and Suriname.

The travel time between the resting site and mine was one day or more for 71.7% of the individuals (208/290). Of the individuals recruited at Ilha Bela, 24 (51.1%) took less than one day to reach the mines, compared to 24% of the individuals in the town of Oiapoque (*p* = 0.0001). Simultaneously, participants mainly used *catraia* (a type of rustic boat usually made of wood) (89, 30.6%) or the route on foot (77, 26.5%). Other means cited were boats (44, 15.1%), cars (12, 4.1%), and planes (3, 1.0%) (Supplementary Table S1).

During their time working in the mines, 50.3% (146/290) of the individuals had worked for more than 10 years, with 20 days as the minimum and 40 years as the maximum. The median time working in the mines was 10 years (IQR: 3.5–16), with statistically significant differences between men and women; the median time working in the mines among men was 11 years (IQR: 5–17), and among women, 5 years (IQR: 2–10.3), *p* = 0.0014. A positive correlation was also found between age and length of time working in the mine (Spearman: 0.58 95% CI: 0.50–0.66, *p* < 0.0001). Among those under 21 years of age, the median time working in the mines was 5 months (IQR: 3 months to 1 year, maximum 7 years). For those between 21 and 40 years of age, this median was 7 years (IQR: 2–10 years, maximum 26 years). For those over 40 years of age, the median was 15 years (IQR: 8–30, maximum 40 years; *p* < 0.0001); 24 (83%) individuals worked for ≥25 years as gold miners. No differences were found in the median time spent working as garimpeiros between those from Ilha Bela and Vila Brasil and those from Oiapoque (*p* = 0.224).

**Figure 4:**
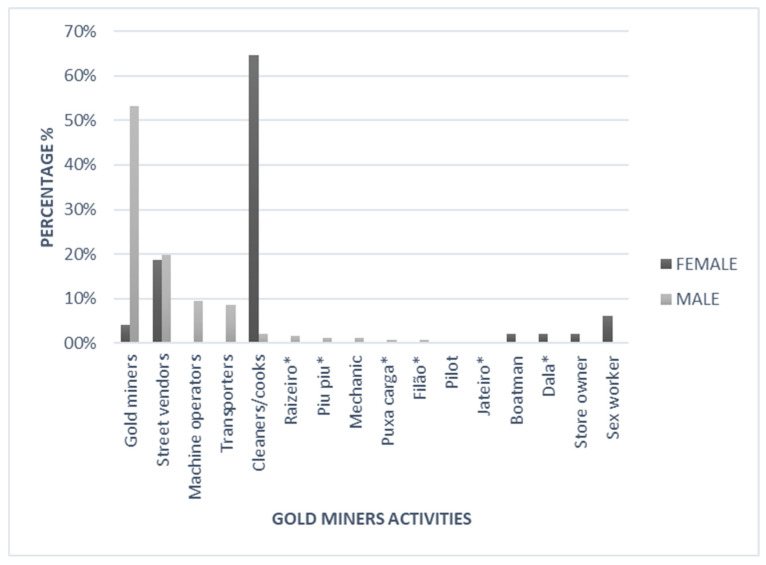
Activities carried out by garimpeiros in the mining sites according to sex. * Raizeiro: Responsible for removing the roots of plants and trees to excavate the ravine. Piu Piu: Name given to the person who uses a magnetic detector in the mines in search of gold. Puxa Carga: Carrier of cargo and materials into the gold mines. Filão: Responsible for opening the trench where the gold will be sought. Jateiro: Responsible for washing the gold and mercury. Dala: Individual who is responsible for making the steps as the wells are dug.

A total of 16 different work activities were identified in the mine. Most were dedicated to mining or excavating the earth looking for gold (garimpeiros stricto sensu, s.s) (131/291, 45.0%), followed by sellers (57/291, 19.6%), cleaners/cooks (36/291, 12.4%), machine operators (23, 7.9%), and transporters (21, 7.2%). Among women, 64.5% (31/48) reported being domestic workers/cookers, whereas among men, 53.1% (129/243) were garimpeiros, 19.7% (48/243) were sellers, and 9.4% (23/243) were machine operators. Only one woman reported being a sex worker. The other activities are shown in Figure 4. Most garimpeiros (69.3%; 201/290) reported working only during the day, and 29.6% (86/290) worked both day and night. No significant differences were found between men and women regarding their work regimes.

This population usually spent a lot of time inside the mine in the forest; the median number of times they left the mine in the last six months was one (IQR: 1–3) time with a maximum of 30 times. In total, 168 (approximately 60%) miners had not left or only left the mine once in the last six months, but 58 (20.7%) had left the mine at least four times. Participants aged 41–60 years (39.7%) usually spent most of their time without leaving the mine. On the first visit, the median departure was 1.0 (IQR: 1–2) times, whereas on the second visit, the median number was 2.0 (IQR: 1–3; *p* = 0.022). The reasons for leaving the mines were diverse, ranging from family visits or rest (51.0%, 147/288) or medical treatment (19.8%, 57/288), and 12.5% (36/288) of the cases were for logistical or financial matters (to buy new materials to sell in the mines or for having received a good amount of gold and having to leave in a rush). Another reason cited (9.4%, 27/288) was that the French police were conducting operations to combat mining activities, and the participants had to leave in haste to escape (Supplementary Table S1). Among those who used to leave, they frequently went to more than one city: Oiapoque (67.8%), Macapá (36.6%), or Belém (14.3%), were the most visited ones (Figure 2B). In addition to Brazil, Cayenne in French Guiana was the main destination (9.8%), followed by Paramaribo in Suriname (5.1%).

### 3.3. Garimpeiro’s Health Status Perceptions

Several health problems that occur inside the mines were mentioned; 71.6% (209/292) reported malaria as the main problem, with 75.9% (101/133) of the miners who were in the Camopi and Haut-Approuague area and 55.6% (89/160) of those from the other mines reporting as such; these differences were statistically significant (*p* = 0.0002). Leishmaniasis was the second most frequent problem cited by garimpeiros (61%; 178/292). Unlike malaria, no areas where this disease was more frequent were found. Other health problems mentioned were flux symptoms (15.8%, n = 46), musculoskeletal problems (10.9%, n = 32), headaches 11.4%, (34), and digestive problems (6.1%, n = 18). Other related diseases included skin diseases (including mycosis; 6.5%, 19/292), bat bites (1.3%, 4/292), accidents (1.3%, 4/292), hepatitis (2%, 6/292), anemia (3.1%, 9/292), and HIV infection (1.7%, 5/292). When further evaluating the causes of digestive problems, a significant number of garimpeiros related gastritis, gastric ulcer, and gastrointestinal infections, including “amoebiasis” (without any parasitic diagnostic). Accidents caused by machinery in the mines have also been reported. One participant reported hunger as a significant problem. Only 14 individuals (4.8%) reported no health problems in the mines (Table 2).

### 3.4. Diseases History

The questionnaire had specific questions about some diseases known to affect gold miners, such as malaria and acquired immunodeficiency syndrome virus (AIDS/HIV) infection. 

Malaria was the disease most reported by the mining community (83.9%, 245/292); of these, 37.5% (92/245) had between one and three previous episodes; 11.8% (29/245) had experienced it between four and seven times, and 50.6% (124/245) more than seven times. A total of 30 (10.4%) individuals reported taking antimalarial drugs in the mine, in the month before the interview. Of these, 16 (53.3%) took some combination therapy with artemisinin derivatives. Four took chloroquine alone or combined with primaquine, and three did not remember. Further, another participant, who was interviewed on the second visit, used Malakit. Additionally, 18 (10.4%) participants did not know whether they had any history of contact with HIV and AIDS, and one (0.5%) claimed to have had some contact with the virus. Of this population, 60 (50.8%) participants had already been tested for HIV and had negative results; three (2.5%) did not want to answer.

### 3.5. Clinical Examination

Only seven participants (2.7%) were feverish during the clinical examination, six (3.4%) from the first cross-sectional study, and only one (0.8%) from the second cross-sectional study; the latter was positive for *Plasmodium vivax* infection in the thick smear and PCR. Participants were asked about fever 48 h before the interview only in the second survey, in 2019; seven individuals (6.9%) answered affirmatively, of which two (28.5%) were positive for malaria (*P. vivax*) in the thick smear and PCR; three (2.5%) individuals had mild jaundice, one of which was positive for *P. vivax*, and 24 (20.3%) had signs of hepatomegaly. In total, 3.4% (10/292) of the individuals were infected with *Plasmodium* spp. Of them, nine were infected with *P. vivax* and one with *P. falciparum*.

A total of 20 individuals (6.8%) had splenomegaly upon clinical examination. In the first visit, 8.0% (14/174) of individuals examined presented splenomegaly; seven (50%) were grade I, six (42.8%) were grade II, and one (7.1%) was grade III. Of these, one individual with grade I splenomegaly was infected with *P. vivax*, and one with grade III splenomegaly was infected with *P. falciparum*, which was positive in the thick smear and PCR. In 2019, of a total of 118 individuals, six (5.0%) had some degree of splenomegaly, four (66.6%) were grade I, and two (33.3%) were grade II; of these, one grade I and one grade II individual were positive for *P. vivax* in the thick smear and PCR. These differences were not statistically significant (*p* = 0.34).

A total of 52 individuals (18.2%) were diagnosed with High Blood Pressure (HBP) at the time of clinical examination, of which 36 (13.1%) had grade I, 2 (0.7%) had grade II, and 14 (5.1%) had isolated systolic pressure. The median age of the hypertensive participants was 48 years (IQR: 38–54), and the median age of the normotensive participants was 37 years (IQR 28–46); these differences were statistically significant (*p* = 0.0001). Surprisingly, we found that 30.8% (16/52) of hypertensive individuals were under 40 years old. Men and women had no differences regarding the inclusion site or visit.

The median BMI among all participants was 22.1 (IQR: 20.2–24.8), with the minimum being 13.5 (underweight) and the maximum 34.9 (obesity); 26 (10.6%) individuals were classified as underweight and 13 (5.3%) with obesity. The median BMI among hypertensive individuals was 24 (IQR: 21.5–26.20), while the median BMI among normotensive people was 21.8 (IQR: 19.65–23.90). These differences were significant (*p* = 0.0007). No differences were found between the sexes.

In total, 35 individuals (12.1%) had ulcers or scars compatible with cutaneous leishmaniasis. In the first visit, 26 (15.1%) participants had active, or scarring from, leishmaniasis-related skin ulcers. Four of them (15.4%) had active lesions with a raised border, compatible with leishmaniasis; 21 (12.2%) participants said they had taken Pentacarinat^®^ (pentamidine isethionate) or Glucantime^®^ (meglumine antimoniate), drugs used to treat leishmaniasis in the last year. On the second visit, nine (7.6%) individuals had a lesion suggestive of a leishmaniasis scar; eight (6.7%) had taken Pentacarinat^®^ or Glucantime^®^ in the previous year. Analyzing by activity carried out in the garimpo, it was observed that the garimpeiros (s.s) were three times more likely to have had leishmaniasis than the other occupations (*p* > 0.01) (Table 3).

## 4. Discussion

Over the last 30 years, thousands of Brazilian artisanal and small gold miners (ASGM) have irregularly crossed the international border between Brazil and French Guiana to work in the mines of the Guiana Shield [6,9]. Despite this intense traffic, there is little information on the sanitary characteristics, living conditions, and illness risks of ASGM crossing the border in Oiapoque. Most garimpeiros result from social marginalization and the lack of a fair and structured rural policy; many people that come from impoverished rural communities find mining to be their only economic option [18]. Our results showed that 87% of gold miners came from Maranhão, Pará, and Amapá, which are among the top five countries in terms of poverty rate [7,9,17,19]. Our study also showed that 74% of the gold miners were either illiterate or had only finished elementary school, highlighting the vulnerability of these populations when it comes to finding steady employment in their place of origin. Women have a higher level of education than men, and data from the Brazilian National Statistical Authority showed the same pattern in the general national population [20].

Our population mainly comprised adult men with a median age of 38 years. Similar results were found by Douine et al. [7] on the border between French Guiana and Suriname and Becker et al. [5] in Zimbabwe. A study conducted during the COVID-19 pandemic showed that 25% of the individuals working in mines in Suriname and 20% working in French Guiana were women [3]. We found that less than 20% of mining community in this area are women, and most of them had been working for less time in the mines (median of 5 years) compared to men, as was also observed in other areas of Brazil [21], Suriname [22], and French Guiana [7]. 

### 4.1. Life in the Mines

The number of mines visited in the last three years by the participants in our study was higher than 60 different sites, which is quite worrying because the exploitation of these areas generates significant deforestation with a robust environmental impact [18]. Deforestation caused by mining activities may create, modify, or increase the larval habitats already existing in these sites due to the opening of the forest itself and favoring of the creation of pools, facilitating an increase in the vector population of different infectious agents, including malaria [22,23,24,25,26]. We found differences in the mines where gold miners worked according to the place of inclusion. Ilha Bela and Vila Brasil are located more than five hours west of Oiapoque and can only be reached after a fast boat trip along the Oiapoque River, which takes between 5 and 7 h. Although these resting places are further away from the city, they are less than a day away from the mines located in the southwest and west of French Guiana, making it easier to return for shopping and other activities. 

Conversely, individuals recruited in the city of Oiapoque prefer to work in the mines to the east or north of French Guiana, where they arrive after more than a day of travel. These miners travel in a dangerous journey along the Atlantic Sea to enter the Maroni River and reach the mines on the border with Suriname.

Mining is a structured activity with well-defined functions. Studies have pointed out various occupations within the same mine from the 1990s [27] until today [7,28], from the owners of the mine to those who often do not work directly in the activities of the mine itself but help in the “financing” of it, either through machinery or supplies, among others. In this study, numerous occupations ranged from cooks to machine operators and sales individuals. How these activities are carried out within a mine site results in a highly mobile population that tends to migrate, searching for a new mining site or job. Gold miners usually spend most of their time working outdoors; in our study, approximately 30% of the miners worked during the day and at night, which could result in greater exposure to mosquito bites. 

Our results indicate that approximately 50% of the mining community interviewed had been working in this activity for more than ten years and usually left the mines only on specific occasions, such as seeking medical attention, and often only because of police operations were being conducted in search of irregular mines. Our results show that only 14.7% of the gold miners interviewed stayed in the mines without leaving in the 6 months before the study. These data are consistent with information indicating that in 2020, only 11.2% of the gold miners in French Guiana remained in these mines for the last year, whereas 43.2% of the Suriname workers remained there. This longer stay in the mines in Suriname can be explained by the miners’ feelings of “being at home” in this country compared to the instability in French Guiana due to the gendarmerie’s fight against irregular mining [3]. Among those who used to leave, they frequently went to more than one city: Oiapoque (67.8%), Macapá (36.6%), or Belém (14.3%) were the most visited ones. Beyond Brazil, Cayenne in French Guiana was the leading destination of interviewees (9.8%), followed by Paramaribo (5.1%). This information differs from the studies of Heemskerk et al. [3] and Douine et al. [7], who included individuals on the border between French Guiana and Suriname. Notably, most individuals who leave for Paramaribo exhibit different behaviors depending on their location of inclusion of the participants. Although access to healthcare facilities for emergencies is free on both sides of the border (French Guiana and Brazil), factors such as the distance to mining areas, the high cost of transportation to leave these locations, and sometimes the irregular legal status in which they live make it difficult for this population to seek hospitals and healthcare facilities [4,7].

### 4.2. Malaria and Other Health Problems Associated with This Population

Among the health problems perceived by this population inside the mines, the most frequently mentioned was malaria, and most (84%) of the participants had a previous history of the disease, which was expected because the prevalence of malaria among gold miners is high [9,29,30]. Surprisingly, the gold miners recruited in Ilha Bela and Vila Brasil working in the mines in southwest and west French Guiana had a higher perception of malaria as a significant problem within the mines compared to people included in the town of Oiapoque working in the mines in east French Guiana (*p* = 0.0002). There is a differential risk of malaria according to geographic area, and further analysis should be performed to confirm these findings.

Cutaneous leishmaniasis was perceived as an essential health problem by 42.7% of the garimpeiros in our study, but we did not find differences in perceptions between inclusion sites. Clinical examination showed that 12.2% of the individuals had skin scars compatible with past or present leishmaniasis. Leishmaniasis was the second most common cause of disease in a study by Douine et al. [31] in this setting, accounting for 52.4% of miners, and 8.3% had active scarring during clinical examination. Loiseau et al. [32] found that 59.3% of patients with leishmaniasis in French Guiana mainly were gold miners of Brazilian origin. In 2019, Vasconcelos et al. [33] conducted a study in Oiapoque and reported that 49% of cutaneous leishmaniasis cases occurred in gold miners. Mosquera Atehortua et al. (2024), using part of our samples, showed that 76% of the 105 participants had evidence of *Leishmania* infection when laboratory techniques and the clinical evaluations were done together [34]. The profound environmental modification produced by mining activity leads to strong pressure with subsequent deforestation, disturbing the ecological niches of the phlebotomine sandflies and significantly increasing human contact with the vector [35]. 

A more recent study conducted by Heemskerk et al. [3] on the Suriname border showed that 26.9% of miners believed that COVID-19 was the primary concern, followed by malaria (24.4%) and leishmaniasis (23.3%). As our study was conducted before the pandemic, we did not have data about COVID-19 perceptions, but we believe that it is a vital disease burden in these populations [36]. 

### 4.3. Accidents and Animal Attacks

During the second survey, some participants reported cases of bat attacks that were observed in the same population in other Amazonian regions. In the first months of 2024, three cases of death from human rabies among miners in French Guiana were reported. This is a serious problem whose prevention must be prioritized in these areas [37]. In Maranhão state, in Brazil, in an Amazonian rainforest area, an outbreak of human rabies was described in 2005 with the death of 16 individuals out of 57 (8.7%) of the total population who bats attacked [38]. Douine et al. [38] showed that more than 60% of the garimpeiros recruited in 2022 by the Guiana Shield suffered from bat attacks. Therefore, this risk cannot be minimized, as an outbreak was reported in mines located in southeastern Venezuela, where 154 gold miners out of a population of 1500 suffered from bat attacks [30]. Similar to Leishmania and other zoonotic diseases, deforestation can lead to the loss of typical bat habitats, forcing bats to turn to humans as an alternative food source. This is a matter of current concern, given the increased chances of human encounters with unknown pathogens in wild environments [38]. Other problems that may affect this population have been reported in the literature, such as leprosy [39], hepatitis [40], sexually transmitted infections [40,41], and hantavirus infections [42]. There are also reports of accidents [3,43], including the risk of illness and psychological disorders [44]. 

### 4.4. Potential Health Risk Determinants

Contamination with heavy metals, especially mercury (Hg), is another frequent problem in this population [30]. There are mainly two types of gold exploitation in artisanal and small-scale mining: alluvial, also known as “baixão”, carried out in rivers and streambeds, where the superficial layer of the soil is exploited, and pits being manually excavated for extraction [45]. Alluvial exploitation has the most significant impact (the central exploitation cited in this study), in which the vegetation cover and topsoil are first removed to reach the gold layer. The gold layer is then dismantled with strong water jets, and the areas where the gold particles are found are washed away. Mercury is used to separate the gold [45]. Our study did not assess the population’s exposure to metals commonly used in gold mining. However, this is a danger to human beings and the surrounding ecosystem. In 2001, Fréry [46] showed that 57% of the individuals from an Amerindian population in French Guiana who followed a diet of fish from the Maroni River had mercury poisoning with higher levels (>1.6 µg/kg) than those predicted by the World Health Organization. Furthermore, approximately 14% of the fish collected also had exposure levels above the limit, which correlated with the miners’ activities in the region. In Pará, the risk of mercury exposure was associated with gold mining in the indigenous population of the state [47].

Another critical issue is the risk of High Blood Pressure (HBP). Although the percentage of HBP in our study was 18.2%, less than the national mean in Brazil (22–40%), our population with HBP is younger (median 48 years old) than the average of the Brazilian population with the hypertensive disease that lives in rural areas (≥60 years old) [48]. The fact that we found 30.8% of hypertensive individuals under 40 years old, along with a higher median of BMI among hypertensive individuals, alerts health authorities to carry out actions to promote health and control of this disease, which is an important factor in cardiovascular risk. 

Our research did not delve into sexually transmitted infections (STI) and risk habits. Nonetheless, Mutricy-Hureau et al. [49], working with this population in the frontier with Suriname and Brazil, found that men often resorted to paid sex, and most women in the gold mining sites did not engage in sex work. This study also revealed high percentages of excessive alcohol and tobacco use. 

Finally, owing to the vulnerability and undocumented status of garimpeiros associated with the distance to healthcare centers, self-medication is a common practice. Often, owing to the high price of medications, garimpeiros use smaller doses than necessary or buy counterfeit drugs illegally sold inside the mines. This behavior could threaten the emergence of antibiotic and antiparasitic drug resistance [6,29,50]. Initiatives such as the Malakit Project could be promising strategies for disease control in these specific contexts [10]. This population continues to live amidst the epidemiological transition, still suffering from infectious-parasitic diseases such as malaria and leishmania, and incidents caused by venomous animals and bat attacks, as well as the so-called “progress” diseases such as hypertension. The disease situation, as well as the high vulnerability of the mining community and their limited access to health services, pose an enormous challenge to the health systems in this region. Specific actions must be thought of to improve the health status of these individuals. 

### 4.5. Limitations of the Study

Despite our sampling efforts, we were only able to interview 292 individuals. Part of the difficulty was due to police operations during the second fieldwork period (2019), which made it impossible for this population to leave the mining sites and reach the resting areas. Additionally, military operations created insecurity among the gold miners, making them hesitant to agree to participate in the research. Although the exact size of the population universe is not known, we believe our sample is sufficient to describe this population. Also, since this study required personal responses, there may have been some non-response bias.

## 5. Conclusions

Oiapoque has served as a resting point for individuals working in irregular gold mines in French Guiana. The miners on this border are all Brazilians with low educational levels. This population is exceptionally vulnerable and subjected to heavy routines, besides being exposed to several diseases such as malaria and leishmaniasis, among other health problems that may spread beyond the border regions. Health problems such as STIs, excessive alcohol and tobacco consumption, and risky behaviors must be addressed. Due to the living and mobility conditions of garimpeiros, zoonotic diseases require in-depth studies, as these individuals may be a bridge to urban populations. Other chronic degenerative diseases, such as arterial hypertension and osteoarthrosis, must be better clarified. Knowledge of this is essential for developing public health policies that can be implemented in specific epidemiological situations.

## Figures and Tables

**Table 1:** Sociodemographic data from the garimpeiros of Oiapoque city, Brazil.

Variables	Visit 1 N (%)	Visit 2 N (%)	OR (IC95%)	*p* Value	Total N (%)
Sex					
Male	147 (84.5)	96 (81.4)			243 (83.2)
Female	27 (15.5)	22 (18.6)	1.2 (0.6–2.3)	0.4	49 (16.8)
Total	174 (100)	118 (100)			292 (100)
Age					
<20 y	7 (4.1)	8 (6.9)		1	15 (5.2)
21–40 y	91 (52.9)	60 (51.7)	0.5 (0.1–1.6)	0.3	151 (52.4)
41–60 y	71 (41.3)	45 (38.8)	0.5 (0.1–1.6)	0.2	116 (40.3)
>61	3 (1.7)	3 (2.6)	0.8 (0.1–5.8)	0.8	6 (2.1)
Total	172 (100)	116 (100)			288 (100)
State of origin					
Maranhão	86 (50)	64 (54.2)		1	150 (51.7)
Pará	39 (22.7)	24 (20.4)	0.8 (0.4–1.5)	0.5	63 (21.7)
Amapá	25 (14.5)	17 (14.4)	0.9 (0.4–1.8)	0.7	42 (14.5)
Other	22 (12.8)	13 (11.0)	0.7 (0.3–1.6)	0.5	35 (12.1)
Total	172 (100)	118 (100)			290 (100)
Level of education					
None	27 (15.5)	11 (9.3)		1	38 (13)
Primary	108 (62.1)	72 (61.0)	1.6(0.7–3.5)	0.2	180 (61.6)
Secondary	38 (21.8)	34 (28.8)	2.1 (0.9–5.0)	0.06	72 (24.7)
Graduation	1 (0.6)	1 (0.9)	2.4 (0.1–42.8)	0.5	2 (0.7)
Total	174 (100)	118 (100)			292 (100)

**Table 2:** Health problems within the mines cited by the population of garimpeiros in the municipality of Oiapoque, Brazil.

Health Problems	Visit 1 N (%)	Visit 2 N (%)	OR (IC95%)	*p* Value	Total
Malaria Leishmaniasis Flu	125 (71.8)	84 (71.1)	-	1	209 (71.6)
	111 (63.7)	67 (56.7)	0.8 (0.5–1.3)	0.6	178 (61)
	39 (22.4)	7 (5.9)	0.2 (0.1–0.6)	0.001	46 (15.7)
Headaches Musculoskeletal problems	23 (13.2)	9 (7.6)	0.5 (0.2–1.3)	0.1	32 (11)
	22 (12.6)	15 (12.7)	1.0 (0.4–2.0)	0.9	37 (12.7)
“Kidney pain” Digestive problems None Skin problems	17 (9.7)	15 (12.7)	1.3 (0.6–2.7)	0.4	32 (11)
	13 (7.4)	4 (3.3)	0.4 (0.1–1.4)	0.1	17 (5.8)
	0 (0.0)	14 (11.8)	-	0.00001	14 (4.8)
	6 (3.4)	13 (11.0)	3.2 (1.1–8.8)	0.01	19 (6.5)
Anemia Myiasis	6 (3.4)	5 (4.2)	1.2 (0.3–4.1)	0.7	11 (3.8)
	3 (1.7)	5 (4.2)	2.4 (0.5–10.6)	0.2	8 (2.7)
HIV	3 (1.7)	3 (2.5)	1.4 (0.2–7.5)	0.6	6 (2.1)
Diarrhea	3 (1.7)	3 (2.5)	1.4 (0.2–7.5)	0.6	6 (2.1)
Hepatitis	3 (1.7)	3 (2.5)	1.4 (0.2–7.5)	0.6	6 (2.1)
Accidents	3 (1.7)	1 (0.8)	0.4 (0.0–4.8)	0.5	4 (1.4)
Chikungunya	3 (1.7)	1 (0.8)	0.4 (0.0–4.8)	0.5	4 (1.4)
Bat attacks	3 (1.7)	1 (0.8)	0.4 (0.0–4.8)	0.5	4 (1.4)
Fatigue	2 (1.1)	2 (1.6)	1.4 (0.2–10.7)	0.6	4 (1.4)
Amoeba	2 (1.1)	0 (0.0)	-	0.2	2 (0.7)
Fever	2 (1.1)	0 (0.0)	-	0.2	2 (0.7)
Yellow fever	2 (1.1)	0 (0.0)	-	0.2	2 (0.7)
Dengue	1 (0.5)	5 (4.2)	7.4 (0.8–64.8)	0.03	6 (2.1)
Stroke	1 (0.5)	2 (1.6)	2.9 (0.2–33.3)	0.3	3 (1)
Hunger	1 (0.5)	1 (0.8)	1.4 (0.0–24.1)	0.7	2 (0.7)
Hypertension	1 (0.5)	1 (0.8)	1.4 (0.0–24.1)	0.7	2 (0.7)
Poluted water	1 (0.5)	0 (0.0)	-	0.4	1 (0.3)
Vomit	1 (0.5)	0 (0.0)	-	0.4	1 (0.3)
Scorpion sting	0 (0.0)	1 (0.8)	-	0.2	1 (0.3)
Pneumonia	0 (0.0)	1 (0.8)	-	0.2	1 (0.3)
Liver problems	0 (0.0)	1 (0.8)	-	0.2	1 (0.3)
Leprosy	0 (0.0)	1 (0.8)	-	0.2	1 (0.3)
Helmintiasis	0 (0.0)	1 (0.8)	-	0.2	1 (0.3)

**Table 3:** Health status of the participants compared by activity carried out in the gold mining site.

Variables	*Garimpeiros * (s.s)	Other Activities	OR (IC95%)	*p* Value
	N (%)	N (%)		
**FEVER**				
Yes	8 (6.6)	5 (3.6)		1
No	114 (93.4)	135 (96.4)	1.8 (0.6–5.9)	0.2
**TOTAL**	122 (100)	140 (100)		
**SPLENOMEGALY**				
Yes	5 (4.1)	15 (10.1)		1
No	117 (95.9)	134 (89.9)	0.3 (0.1–1.0)	0.06
**TOTAL**	122 (100)	149 (100)		
**HIGH BLOOD PRESSURE**				
Yes	27 (21.3)	26 (17.6)		1
No	100 (78.7)	122 (82.4)	1.2 (0.6–2.3)	0.4
**TOTAL**	127 (100)	148 (100)		
**ANY LESION OF LEISHMANIASIS**				
Yes	25 (19.2)	10 (6.4)		1
No	105 (80.8)	147 (93.6)	3.5 (1.6–7.5)	0.0009
**TOTAL**	130 (100)	157 (100)		
**POSSIBLE JAUNDICE**				
Yes	2 (1.6)	4 (2.6)		1
No	122 (98.4)	150 (96.1)	0.6 (0.1–3.4)	0.5
Dubious	0 (0.0)	2 (1.3)		0.3
**TOTAL**	124 (100)	156 (100)		
**HIV ANTECEDENT**				
Yes	25 (19.1)	36 (22.5)		1
No	98 (74.8)	111 (69.4)	0.7 (0.4–1.4)	0.4
Did not answer/do not know	8 (6.1)	13 (8.1)	1.1 (0.4–3.1)	0.8
**TOTAL**	131 (100)	160 (100)		
**POSITIVE PCR FOR MALARIA**				
Yes	5 (3.8)	5 (3.1)		1
No	126 (96.2)	156 (96.9)	1.2 (0.3–4.3)	0.7
**TOTAL**	131 (100)	161 (100)		
**MALARIA AS A CONCERN**				
Yes	92 (70.2)	117 (74.1)		1
No	39 (29.8)	41 (25.9)	0.8 (0.4–1.3)	0.4
**TOTAL**	131 (100)	158 (100)		
**DISTANCE TO THE GOLD MINING SITE**				
Less than a day	32 (24.4)	50 (31.4)		1
More than a day	99 (75.6)	109 (68.6)	0.7 (0.4–1.1)	0.1
**TOTAL**	131 (100)	159 (100)		
**TIME IN THE GOLD MINING SITE**				
Less than nine years	60 (45.8)	84 (52.8)		1
More than ten years	71 (54.2)	75 (47.2)	0.7 (0.4–1.1)	0.2
**TOTAL**	131 (100)	159 (100)		
**WORK ROUTINE**				
Only by day	92 (70.8)	109 (68.6)		1
Only by night	1 (0.8)	1 (0.6)	0.8 (0.0–13.6)	0.9
Both	37 (28.4)	49 (30.8)	1.1 (0.6–1.8)	0.6
**TOTAL**	130 (100)	159 (100)		

## Data Availability

The database of this article will be available at ARCA DADOS, which is FIOCRUZ’s institutional data repository.

## Supplementary Materials

The following supporting information can be downloaded at: https://www.mdpi.com/article/10.3390/tropicalmed10010012/s1, Table S1: Characterization of life in the gold mines by the garimpeiros of Oiapoque city, Brasil.
