# Supplementary material for: Brazilian Gold Miners Working Irregularly in French Guiana: Health Status and Risk Determinants
Source: Trop Med Infect Dis. 2024 Dec 31;10(1):12. doi: 10.3390/tropicalmed10010012 (PMC11768861; doi:10.3390/tropicalmed10010012)
Supplement: Supplementary file 1 [file tropicalmed-10-00012-s001.zip › tropicalmed-3146256-supplementary.pdf]

Supplmentary table. Characterization of life in the gold mines by the garimpeiros of Oiapoque city, Brasil.

Variables	Visit 1	Visit 2	OR (IC95%)	P valor	Total
	N (%)	N (%)			N (%)
**Distance from the mine to the resting place**					
<1 day	58 (33.72)	24 (20.34)	**1.99 (1.15-3.44)**	**0.01**	82 (28.3)
>1 day	114 (66.28)	94 (79.66)			208 (71.7)
**Total**	**172 (100)**	**118 (100)**			290 (100)
**Transport to get to the mines**					
Catraia	58 (33.72)	31 (26.27)		1	89 (30.7)
By foot	43 (25)	34 (28.81)	1.47(0.79-2.76)	0.24	77 (26.6)
Boat	38 (22.10)	6 (5.09)	**0.29 (0.11-0.77)**	**0.01**	44 (15.2)
By foot + catraia	15 (8.72)	25 (21.19)	**3.11 (1.43-6.76)**	**0.003**	40 (13.8)
Car	9 (5.23)	3 (2.54)	0.62 (0.15-2.47)	0.49	12 (4.1)
Plane	2 (1,16)	1 (0.85)	0.93(0.08-10.74)	0.95	3 (1)
Others	7 (4.07)	18 (15.25)	**4.81 (1.81-12.76)**	**0.0009**	25 (8.6)
**Total**	**172 (100)**	**118 (100)**			**290 (100)**
**Mining sites visited in the last three years**					
French guiana	135 (78.03)	61 (51.70)	**3.23 (1.94-5.37)**	**0.000003**	196 (67.1)
FG + others	39 (21.97)	57 (48.30)			96 (32.9)
**Total**	**173 (100)**	**118 (100)**			**291 (100)**
**Types of mines**					
Alluvial	35 (20.23)	83 (71.55)		1	118 (40.8)
Well	44 (25,43)	8 (6.90)	**0.07 (0.03-0.17)**		52 (18)
Both	30 (17.34)	22 (18.96)	**0.30 (0.15-0.60)**	**0.005**	52 (18)
Machines	46 (26.59)	0 (0.00)			46 (15.9)
Others	18 (10.41)	3 (2.59)	**0.07 (0.01-0.25)**	**0.000001**	21 (7.3)
**Total**	**173 (100)**	**116 (100)**			289 (100)
**Number of people working in the current mining site**					
<10	39 (22.54)	25 (21.19)		1	64 (22)
11 a 50	31 (17.92)	30 (25.42)	1,50 (0,74-3.07)	0,25	61 (21)
51 a 100	15 (8.67)	17 (14.41)	1.76 (0.75-4.16)	0,19	32 (11)
>100	75 (43.35)	30 (25.42)	0.62 (0.32-1.20)	0.15	105 (36.1)
Don't know	13 (7.52)	16 (13.56)	1.92 (0.79-4.66)	0.14	29 (9.9)
**Total**	**173 (100)**	**118 (100)**			**291 (100)**
**Mining work regime**					
Only by day	111 (64,91)	90 (76,27)		1	201 (69.5)
Only by night	2 (1,17)	0 (0,00)		0.20	2 (0.7)
Both	58 (33.92)	28 (23,73)	0.59 (0.35-1.01)	0.05	86 (29.8)
**Total**	**171 (100)**	**118 (100)**			**289 (100)**
**Time working as a gold miners**					
<10 years	76 (44.19)	68 (57.63)	**0.57 (0.35-0.91)**	**0.01**	144 (49.3)
>10 years	98 (55.81)	50 (42.37)			148 (50.7)
**Total**	**172 (100)**	**118 (100)**			**292 (100)**
**Reasons to leave the mines**					
To rest/family	87 (50.29)	60 (52.17)		1	147 (51)

Medical treatment	29 (16.76)	28 (24.35)	1.40 (0.75-2.58)	0.28	57 (19.8)
Financial/logistical	19 (10.98)	17 (14.78)	1.29 (0.62-2.69)	0.48	36 (12.5)
Police operations	21 (12.14)	6 (5.22)	0.41 (0.15-1.08)	0.06	27 (9.4)
Others	17 (9.83)	4 (3.48)	0.34 (0.10-1.06)	0.05	21 (7.3)
**Total**	**173 (100)**	**115 (100)**			**288 (100)**
